# Melittin Alleviates Oxidative Stress Injury in Schwann Cells by Targeting Interleukin-1 Receptor Type 1 to Downregulate Nuclear Factor Kappa B-Mediated Inflammatory Response In Vitro

**DOI:** 10.7759/cureus.65721

**Published:** 2024-07-30

**Authors:** Ye-ran Mao, Ling-yi Zhu, Ruo-fei Du, Xiao-yu Liu, Zhidan Liu, Li Li

**Affiliations:** 1 Rehabilitation, Baoshan Hospital, Shanghai University of Traditional Chinese Medicine, Shanghai, CHN; 2 Integrative/Complementary Medicine, Shanghai University of Traditional Chinese Medicine, Shanghai, CHN; 3 Pharmacology, Institute of Innovative Chinese Medicine, Shanghai University of Traditional Chinese Medicine, Shanghai, CHN; 4 Respiratory Intensive Care Medicine, Baoshan Hospital, Shanghai University of Traditional Chinese Medicine, Shanghai, CHN

**Keywords:** nf-κb, il-1r1, idiopathic facial paralysis, schwann cell, melittin

## Abstract

Background and objectives: In ancient China, bee venom was widely used to treat various diseases. Although using bee venom is not currently a mainstream medical method, some have applied it to treat certain conditions, including idiopathic facial paralysis (IFP). Recently, melittin (Mel), the main active component of bee venom, has been shown strong anti-inflammatory and analgesic effects. However, how bee venom improves neurological dysfunction in facial paralysis remains unknown. This study aimed to investigate the anti-neurotraumatic effect of Mel on Schwann cells (SCs), the main cells of the neuron sheath, injured by oxidative stress.

Methods: A model of hypoxic SCs was established, and CCK-8 assay, siRNA transfection, enzyme-linked immunosorbent assay, quantitative reverse transcription-polymerase chain reaction, western blot, immunofluorescence, and cell ultrastructure analyses were conducted to investigate the mitigation of hypoxia-induced damage to SCs in vitro, revealing the effects of Mel on oxidative stress injury in SCs.

Results: The overexpression of HIF-1α in CoCl_2_-induced SCs (p < 0.05) indicated the establishment of an SCs hypoxia model. The proliferation and regeneration process of the hypoxic SCs enhanced in the Mel-treated group compared to the CoCl_2_ group has been proven through the CCK-8 experiment (p < 0.0001) and S-100 mRNA expression detection (p < 0.0001). The increased level of reactive oxygen species (ROS) (p < 0.001) and decreased superoxide dismutase (SOD) levels (p < 0.05) in the CoCl_2_-induced SCs indicated that Mel can alleviate the oxidative stress damage to SCs induced by CoCl_2_. Mel alleviated oxidative stress and inflammation in hypoxic SCs by reducing pro-inflammatory cytokines IL-1β (p < 0.0001) and TNF-α (p < 0.0001). In addition, Mel augmented cellular vitality and regulated indicators related to oxygen metabolism, cell repair, neurometabolism, and vascular endothelial formation after hypoxia, such as C-JUN (p < 0.05), glial cell line-derived neurotrophic factor (GDNF; p < 0.001), vascular endothelial growth factor (VEGF; p < 0.05), hypoxia-inducible factor 1-alpha (HIF-1α; p < 0.05), interleukin-1 receptor type 1 (IL-1R1; p < 0.05), enolase1 (ENO1; p < 0.05), aldose reductase (AR; p < 0.01), SOD (p < 0.05), nerve growth factor (NGF; p < 0.05), and inducible nitric oxide synthase (iNOS; p < 0.05). In terms of its mechanism, Mel inhibited the expression of proteins associated with the NF-κB pathway such as IKK (p < 0.01), p65 (p < 0.05), p60 (p < 0.001), IRAK1 (p < 0.05), and increased IKB-α (p < 0.0001). Moreover, knocking out of IL-1R1 in the si-IL-1R1 group enhanced the therapeutic effect of Mel compared to the Mel-treated group (all of which p < 0.05).

Conclusion: This research provided evidence of the substantial involvement of IL-1R1 in oxidative stress damage caused by hypoxia in SCs and proved that Mel alleviated oxidative stress injury in SCs by targeting IL-1R1 to downregulate the NF-κB-mediated inflammatory response. Mel could potentially serve as an innovative therapeutic approach for the treatment of IFP.

## Introduction

Bee venom therapy was once popular among the general public in China, targeting mostly inflammatory and neurological diseases, including idiopathic facial paralysis (IFP). Such therapy is a vivid example of using natural products to treat diseases and serves as a reminder that there may be some scientific truths that our ancestors could not explain. How bee venom treats IFP remains an unanswered question.

IFP is considered a facial inflammatory lesion of uncertain cause whose basic pathology is characterized by demyelinating change, manifesting as facial nerve paralysis, facial nerve innervation muscle, and other target organ dysfunction [1]. The etiology of IFP is thought to be multifaceted. In addition to pathogen infection (especially viral infection, including the novel coronavirus) [2,3] and cytokine dysregulation [4], acute cold exposure [5] is considered another major cause of the disease.

Low temperature can lead to vasoconstriction, abnormal neuroregulation, tissue ischemia and hypoxia, and nerve ischemia and hypoxia, which can then induce oxidative stress [6,7], representing a potentially important intrinsic pathological process of facial nerve injury. Previous transcriptomic studies have detected increased interleukin-1 receptor type 1 (IL-1R1) in patients with Bell’s palsy [8], suggesting that IL-1R1 plays an important role in the occurrence and development of IFP.

In this study, we investigated the effects of melittin (Mel) on the proliferation and apoptosis of Schwann cells (SCs) under oxidative stress and discussed the potential mechanism of IL-1R1 intervention in the nuclear factor kappa B (NF-κB) signaling pathway.

## Materials and methods

Cell culturing and intervention

This experimental study was approved by the Medical Ethics Committee Board of Baoshan Hospital, Shanghai University of Traditional Chinese Medicine.

Commercial rat SCs of the CM-M111 cell line were purchased from Procell Life Science & Technology, China. The SCs were cultured in a SC medium (ScienCell, Carlsbad, CA) comprising a base medium of 5% fetal bovine serum, 1% SC growth supplement, 1% penicillin, and 1% streptomycin. All cells were stored in 5% CO_2_ at 37°C. The SCs were divided into four groups (n = 5 for each group) and allowed to grow to a cell confluent rate of 70%-80%: (1) the negative control (NC) group, (2) CoCl_2_ group, (3) CoCl_2_+Mel group, and (4) CoCl_2_+si-IL-1R1+Mel group.

CoCl_2_ is a commonly used agent to induce hypoxia-like reactions, including preventing degradation, stabilizing hypoxia-inducing factors, and accumulating HIF-1α protein. As per the literature, SCs were cultured in a medium containing 240 μM CoCl_2_ for eight hours under atmospheric conditions (95% air and 5% CO_2_) in a humidified incubator at 37°C (21% O_2_) [9]. Then, in the Mel group, SCs were cultured with Dulbecco's Modified Eagle Medium containing 30 μg/mL Mel (Aladdin, China) for 30 h after siRNA infection or non-infection.

Cell counting kit 8 (CCK-8) detection

SCs activity was determined using CCK-8 reagent according to the manufacturer’s instructions (Biyuntian Biotechnology, China). Cultured SCs were inoculated on a 96-well cell culture plate (5 × 105 cells/well) before adding 100 µL of complete growth medium at 37℃ in a 5% CO_2_ humidified atmosphere. When the cell confluent rate reached 70%-80%, 10 μL of CCK-8 solution was added to each well and incubated at 37℃ for 1 h. The optical density (OD) of the 96-well plate was measured at 450 nm by a microplate reader (BioTek, USA), and cell survival was calculated with the following formula:

$Cell\ survival\ rate = \frac{(Experimental\ group\ -\ Blank\ well)}{(NC\ group\ -\ Blank\ well)} &times; 100\%$.

Four groups were established: NC, NC+CoCl_2_, NC+CoCl_2_+Mel, and NC+CoCl_2_+si-IL-1R1+Mel. The experimental results were corrected with blank holes, in which the absorption value from the spectrophotometer was set to zero at 450 nm (phosphate-buffered saline (PBS) + CCK-8 solution).

siRNA transfection

A plasmid containing the si-IL-1R1 transcript (purchased from Gene Universal, Newark, DE) was transfected into the SCs with Hieff Trans® in vitro siRNA/miRNA transfection reagents according to the manufacturer’s instructions (Yeasen, Shanghai, China). si-IL-1R1 expression was detected by quantitative reverse transcription-polymerase chain reaction (RT-qPCR) 24 h after transfection.

Enzyme-linked immunosorbent assay (ELISA)

According to the requirements of different groups, the supernatant was collected after the end of cell culture time and centrifuged at 25℃ and 4000 rpm for 20 min. Pro-inflammatory cytokines IL-1β and TNF-α, reactive oxygen species (ROS), and superoxide dismutase (SOD) in the extracellular supernatant were quantified with the ELISA technique (BOSTER, China). Relevant reagents were added for testing according to the manufacturer’s instructions. Absorbance was measured at 450 nm using an enzyme-labeler (BioTek, USA).

Western blot

The SCs of each group were incubated for 24 h and harvested for western blot analysis. SC protein extracts were then prepared using a total protein extraction kit (Beyotime, China). All cell proteins were mixed with sodium dodecyl sulfate-polyacrylamide gel electrophoresis (SDS-PAGE) loading buffer (P0015, Beyotime, China) and heated at 98℃ for 5 min. Each protein sample (20 μg/well) was added to SDS-PAGE (10%-12%) and electroplated to polyxylene fluoride (PVDF) membranes (Millipore, Burlington, MA). Each PVDF membrane was closed with 5% skim milk (BD, Franklin Lakes, NJ) at room temperature for 60 min and then incubated overnight with the following primary antibodies at 4℃: anti-HIF‐1α antibody (#14179, Cell Signaling, 1:1000), anti-GAPDH antibody (ab245355, Abcam, 1:1000), anti-VEGF antibody (#9698, Cell Signaling, 1:1000), anti-IL-1R1 antibody (#6775, Cell Signaling, 1:1000), anti-ENO1 antibody (#3810S, Cell Signaling, 1:1000), anti-C-JUN antibody (#9165T, Cell Signaling, 1:1000), anti-glial cell line-derived neurotrophic factor (GDNF) antibody (#47808, Cell Signaling, 1:1000), anti-IRAK1 antibody (#4395, Cell Signaling, 1:1000), anti-IKKα antibody (#2682 Cell Signaling, 1:1000), anti-p65 antibody (ab16502, Abcam, 1:800), anti-p50 antibody (ab305263, Abcam, 1:900), anti-IKB-α antibody (#8943, Cell Signaling, 1:900), anti-NGF antibody (#2046, Cell Signaling, 1:1000), and anti-β-actin antibody (ab179467, Abcam, 1:1000). Each PVDF membrane was then incubated with horseradish peroxidase (HRP)-labeled secondary antibody (MRBiotech, China) at 25°C for 60 min, and signals were collected using Image Studio™ Digits v4.0 (Wroclaw, Poland). Density values were standardized in β-actin or GAPDH. Image-J software (National Institutes of Health) was used to quantify the western blot data.

RNA isolation and RT-qPCR

Total RNA samples were extracted from cultured cells using the TRIzol reagent (R0011, Beyotime, Jiangsu, China) according to the manufacturer’s instructions. RT-PCR for IL-1β and TNF-α, S100, and IL-1R1 was performed using SYBR qPCR Mix (D7260, Beyotime, Jiangsu, China). A 20-μL reaction system was established according to the manufacturer’s instructions, and amplification took place over 40 cycles. Expression levels were standardized by β-actin. RT-PCR was performed on an RT-PCR apparatus (Tannon1600, Shanghai, China). Finally, melt curve analysis was performed to verify the specificity of the expected PCR products. The relative expression is calculated using the 2[−∆∆Ct] method. Five independent samples were prepared for each assay, and each experiment was performed three times. Primer names and primer sequences are listed in Table 1.

**Table 1:** Primer sequences

Gene	Primer sequences (5’-3’)
IL-1β	Forward 5’-TTCAGGCAGGCAGTATCACTC-3’
	Reverse 5’-GAAGGTCCACGGGAAAGACAC-3’
TNF-α	Forward 5’-CCACGCTСTТСТGТСТАСТG-3’
	Reverse 5’-GCTACGGGCTTGTCACTC-3’
S100	Forward 5’-TGGCCCTCATCGACGTTTTC-3’
	Reverse 5’-ATGTTCAAAGAACTCGTGGCA-3’
IL1-R1	Forward 5’-CTGCTGTCGCTGGAGATTGAC-3’
	Reverse 5’-TTGGCAGGTACAAACCAAAGAT-3’
β-actin	Forward 5’-CTCCATCCTGGCCTCGCTGT-3’
	Reverse 5’-GCTGTCACCTTCACCGTTCC-3’
SOD	Forward 5’-GGCTTCTCGTCTTGCTCTCTC-3’
	Reverse 5’-TTCTGCTCGAAGTGGATGGTT-3’
AR	Forward 5’-ATCTGCTGCGTATTGTGGCT-3’
	Reverse 5’-TTTGCTTGGTTGGCACACAG-3’
NGF	Forward 5’-GGGAGCGCATCGAGTTTT-3’
	Reverse 5’-CTGCGGCCAGTATAGAAAGC-3’
iNOS	Forward 5’-GTTCTCAGCCCAACAATACAAGA-3’
	Reverse 5’-GTGGACGGGTCGATGTCAC-3’
IKK	Forward 5’-CCATCGAGACCTGAAGCCAG-3’
	Reverse 5’-CTGATCCAGCTCCTTGGCAT-3’
p65	Forward 5’-GTCGCGCACCTGCTCTC-3’
	Reverse 5’-GTAAAGCCATTCGCCAGAGG-3’
P50	Forward 5’-ACAACTATGAGATGAACTCCGGG-3’
	Reverse 5’-CCGTGGGGCATTTTGTTCAG-3’
IKB-α	Forward 5’-CAGGAGCCAAAACCGACAAC-3’
	Reverse 5’-TGGTTGTCAGGTCTGCAATTT-3’
IRAK1	Forward 5’-CCAGAGGCAAAACTCCCAACA-3’
	Reverse 5’-AGCAGCAGCCCTTTACCACT-3’

Immunofluorescence

The protocol described by Zhao et al. [10] was followed for localization of IL-1R1 in the SCs. Briefly, cells cultured on coverslips were fixed with 4% paraformaldehyde in PBS for 10 min and then permeabilized with 0.05% Triton X-100 for 10 min. Non-specific binding sites were blocked with 5% goat serum for 1 h. Subsequently, the cells were incubated with a rabbit anti-rat IL-1R1 monoclonal antibody (AF7212, Beyotime, Jiangsu, China) at room temperature for 1.5 h. After washing three times with PBS, the cells were incubated with FITC-conjugated goat anti-rabbit IgG (#7074, Cell Signaling) for 2 h at room temperature. Finally, positive cells were observed and quantified using a fluorescence microscope (Nikon, Tokyo, Japan).

Ultrastructure analysis

According to the work of Zhao et al. [10], the SCs were fixed in 2.5% glutaraldehyde and treated with 1% osmium tetroxide overnight at 4℃ (#20816-12-0, Sinma, Singapore). The cells were then dehydrated using ethanol and acetone. Ultrathin sections (80 nm) were made and stained with uranyl acetate and lead citrate (Aladdin, Shanghai, China). Transmission electron microscopy (TEM, HT7800, Hitachi, Japan) was utilized to observe the ultrastructure of the SCs.

Statistical analysis

Group comparisons among more than three groups were analyzed using a one-way analysis of variance with Tukey multiple comparisons via GraphPad Prism v8.0 (GraphPad Software Inc., California). p-values of <0.05 were considered statistically significant, with error bars representing the standard deviation.

## Results

Mel improves oxidative stress injury of SCs caused by CoCl_2_ in vitro

**Figure 1:**
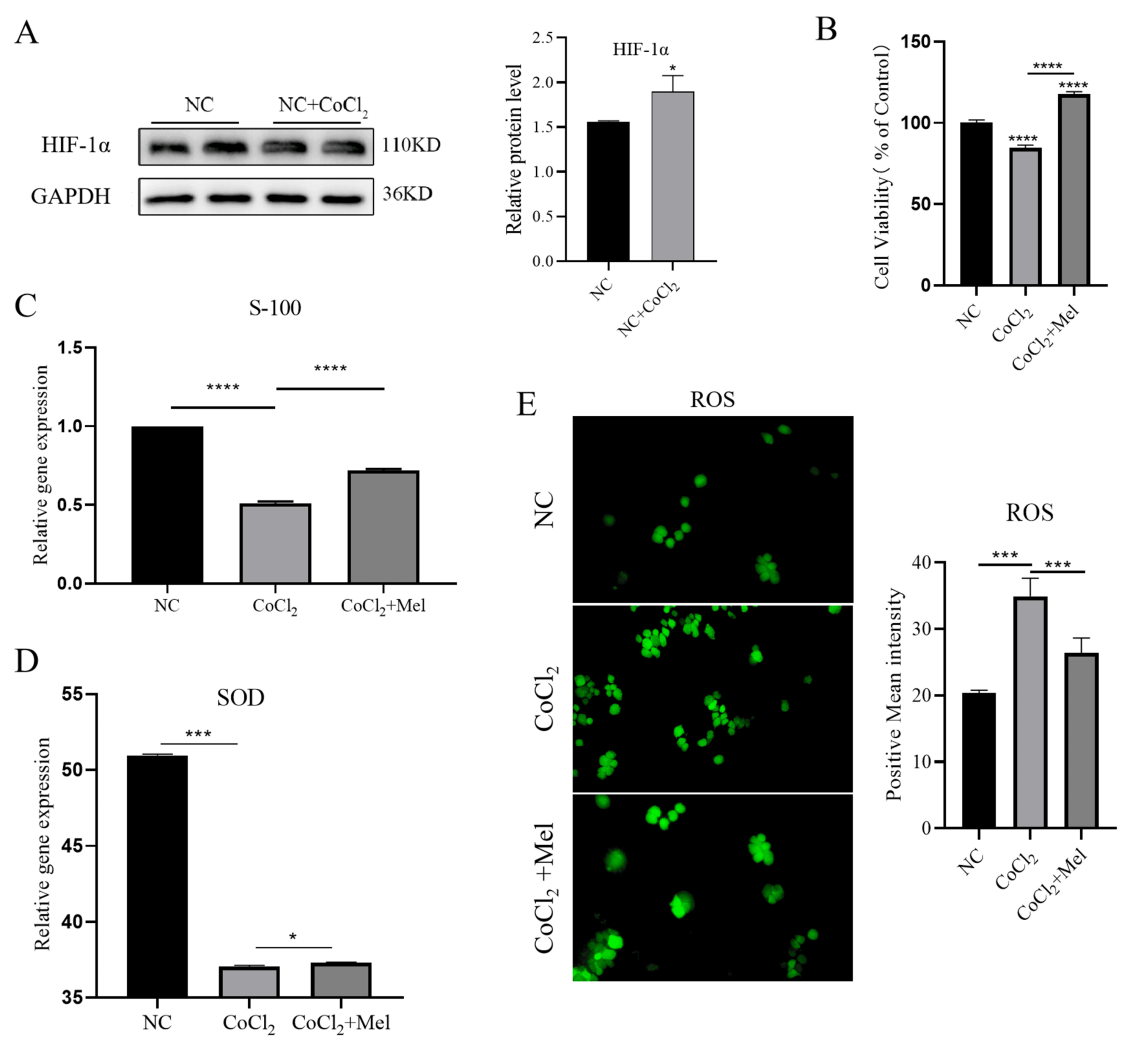
Detection of CoCl2-induced oxidative stress injury in SCs and the intervention effect of Melittin (Mel) (A) Representative western blot analysis of HIF-1α from SCs induced with CoCl_2_ (240 μM) for 8 h. Left and right panels show images and quantitative analyses of HIF-1α in the negative control (NC) and NC+CoCl_2_ groups, respectively. (B) CCK-8 analysis of SCs viability after Mel treatment. (C) Reverse transcription-polymerase chain reaction (RT-qPCR) analysis of mRNA expression of S-100. (D) and (E) ELISA analyses of detected protein expressions of reactive superoxide dismutase (SOD) and reactive oxygen species (ROS), respectively. *p < 0.05. **p < 0.01, ***p < 0.001, ****p < 0.0001 versus control group. Data are presented as mean ± standard deviation. SCs: Schwann cells; HIF-1α: Hypoxia-inducible factor 1-alpha; CCK-8: Cell counting kit 8; ELISA: Enzyme-linked immunosorbent assay.

An SC hypoxia model was established by the administration of CoCl_2_; the hypoxic state of the model was proved through the overexpression of HIF-1a, which served as the hypoxia marker. After the SCs were treated with CoCl_2_, the OD of western blot bands was enhanced, indicating that the hypoxia model was successfully constructed (Figure 1, Panel A). Then, Mel was used to treat hypoxic SCs to evaluate its effects on cell proliferation and the secretion of stress-related proteins. The CCK-8 experiment demonstrated that Mel significantly enhanced the proliferation of the hypoxic SCs (Figure 1, Panel B). To determine the effect of Mel on the SCs regeneration process, RT-PCR analysis was performed to measure S-100 levels. The results indicated that the mRNA expression of S-100 was dramatically decreased in response to the simulated hypoxic environment induced by CoCl_2_. The administration of Mel resulted in the restoration of S-100 expression (Figure 1, Panel C). ROS and SOD are widely regarded as key components of oxidative stress. In the context of cell biology, it is worth noting that SOD plays a key role as the main enzyme responsible for clearing ROS and mitigating the harmful effects of oxidative stress. In addition, we observed that ROS expression levels were upregulated, and SOD protein levels were downregulated under the simulated hypoxia mode. Compared with the NC group, ROS and SOD levels in the CoCl_2_-induced SCs increased and decreased, respectively. Thus, Mel reversed the expression patterns of ROS and SOD (Figure 1, Panels D, E). In summary, Mel can alleviate the stress damage to SCs induced by CoCl_2_, representing potential pharmaceutical value.

Mel reduces the secretion of inflammatory cytokines in SCs under oxidative stress

**Figure 2:**
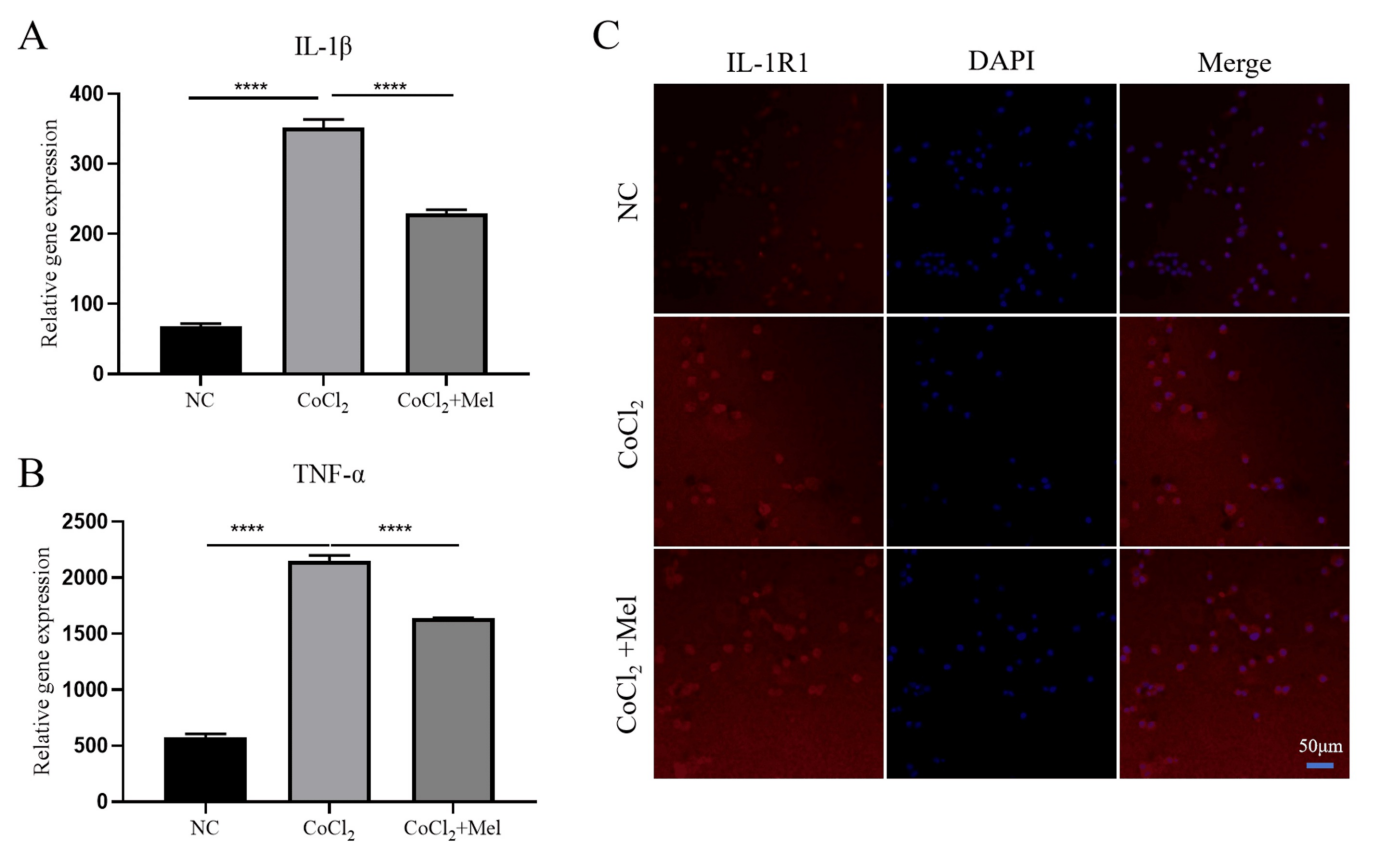
Inflammatory cytokines secretion in hypoxic SCs after Mel intervention (A) and (B): RT-qPCR analysis of reactive mRNA of IL-1β and TNF-α expression in hypoxic SCs and the intervention effect of melittin. (C) Immunofluorescence assays after staining with anti-Fn and anti-IL-1R1 antibodies (scale bars = 50 μm). ****p < 0.0001 versus the control group. Data are presented as mean ± standard deviation. SCs: Schwann cells; RT-qPCR: Quantitative reverse transcription-polymerase chain reaction; IL-1R1: Interleukin-1 receptor type 1; TNF-α: Tumor necrosis factor-alpha; DAPI: 4′,6-diamidino-2-phenylindole.

Based on our previous findings, we propose that Mel has a protective effect on SCs via the inhibition of inflammatory factor production. The mRNA expressions of IL-1β and TNF-α were detected by RT-PCR to confirm this hypothesis. The results showed that the expression of inflammatory factors IL-1β and TNF-α increased in response to hypoxic conditions. In addition, it was observed that Mel had a notable suppressive impact on the mRNA expressions of IL-1β and TNF-α (Figure 2, Panels A, B).

We then attempted to find any correlation between Mel and IL-1β to reveal the mechanism of Mel on CoCl_2_-induced SCs. The location and quantification of IL-1R1 (receptor for IL-1β) were analyzed using immunofluorescence. The results showed that IL-1R1 was expressed on the cell membrane of the SCs, and its expression level was upregulated in response to hypoxia, which was consistent with the trend of IL-1β. After treatment with Mel, there was a significant reduction in the expression level of IL-1R1, as shown in Figure 2, Panel C, which suggested that Mel protected the SCs from damage induced by CoCl_2_ by downregulating the expression of IL-1R1.

Mel-regulated indicators related to oxygen metabolism, cell repair, neurometabolism, and vascular endothelial formation after hypoxia by inhibiting IL-1R1 in CoCl_2_-induced SCs

**Figure 3:**
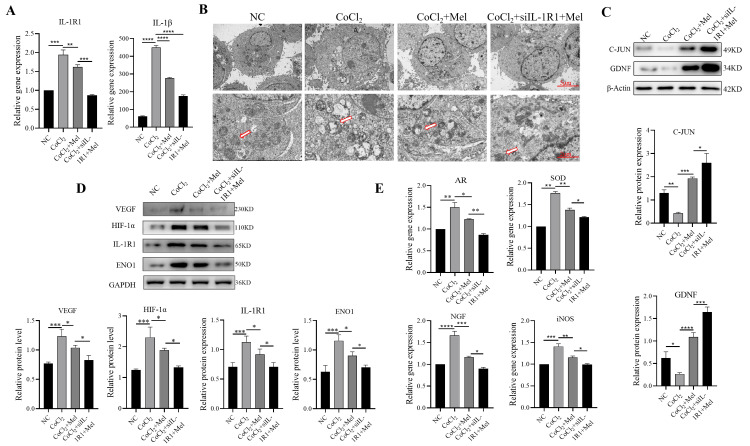
Repair effect of Mel on CoCl2-induced oxidative stress injury and the role of IL-1R1 (A) RT-qPCR results showing the expression levels of IL-1R1 and IL-1β. (B) Transmission electron microscope images of ultrastructural alterations in CoCl_2_-induced SCs for each group (Left: ×2500, Right: ×7000), with the red arrows indicating mitochondria. (C) Western blot of C-JUN and GDNF protein expression for quantitative analyses. (D) and (E) Western blot and RT-qPCR results showing the expression levels of VEGF, HIF-1α, IL-1R1, ENO1, AR, SOD, NGF, and iNOS. *p < 0.05, **p < 0.01, ***p < 0.001, ****p < 0.0001 versus control and other groups. Data are presented as mean ± standard deviation. SCs: Schwann cells; GDNF: Glial cell line-derived neurotrophic factor; RT-qPCR: Quantitative reverse transcription-polymerase chain reaction; VEGF: Vascular endothelial growth factor; HIF-1α: Hypoxia-inducible factor 1-alpha; IL-1R1: Interleukin-1 receptor type 1; ENO1: Enolase 1; AR: Aldose reductase; SOD: Superoxide dismutase; NGF: Nerve growth factor; iNOS: Inducible nitric oxide synthase.

We transfected SCs with si-IL-1R1 RNA to silence IL-1R1 and verify its role and the expression of IL-1β. When Mel was applied to treat SCs with knockdown IL-1R1, RT-qPCR showed that the expression levels of IL-1R1 and IL-1β in the CoCl_2_+si-IL-1R1+Mel group were lower than that in the CoCl_2_+Mel group (Figure 3, Panel A).

The ultrastructure of SCs was examined using TEM (Figure 3, Panel B), which demonstrated that in the control group, the distribution of cell chromatin was uniform, showing no signs of disintegration or pyknosis. Nevertheless, in the group subjected to CoCl_2_ induction, there was an observed reduction in cell body size, condensation of the cytoplasm, and impairment of the mitochondria. In contrast, the treatment group receiving Mel and the treatment group with IL-1R1 knockdown followed by Mel therapy exhibited slight condensation of chromatin, preservation of the nucleoli, and intact nucleolar membranes. In summary, it may be concluded that the administration of Mel therapy has the potential to safeguard impaired SCs via the inhibition of IL-1R1 expression.

C-JUN is a major marker of post-injury SCs, called “repair Schwann cells,” and it plays a key role in facilitating direct communication between SCs and developing nerve protrusions. GDNF is a neurotrophic factor that is actively released by SCs and facilitates the preconditioning process of SCs after peripheral nerve damage. As shown in Figure 3, Panel C, the western blot results demonstrated that the expression levels of C-JUN and GDNF were notably increased in the IL-1R1 knockdown group when compared to the Mel group.

In addition, indicators reflecting neurometabolism and vascular endothelial formation after hypoxia such as vascular endothelial growth factor (VEGF), enolase1 (ENO1), aldose reductase (AR), SOD, nerve growth factor (NGF), and inducible nitric oxide synthase (iNOS) increased significantly after hypoxia (Figure 3, Panels D, E). In contrast, Mel intervention effectively reduced their expressions, and IL-1R1 knockdown by si-IL-1R1 RNA could further reduce their expression to the same levels as those of the NC group. By integrating the findings above, it can be inferred that the administration of Mel has the potential to safeguard SCs by suppressing the production of IL-1R1.

NF-κB pathway mediates the effect of Mel on IL-1R1 expression in CoCl_2_-induced SCs

**Figure 4:**
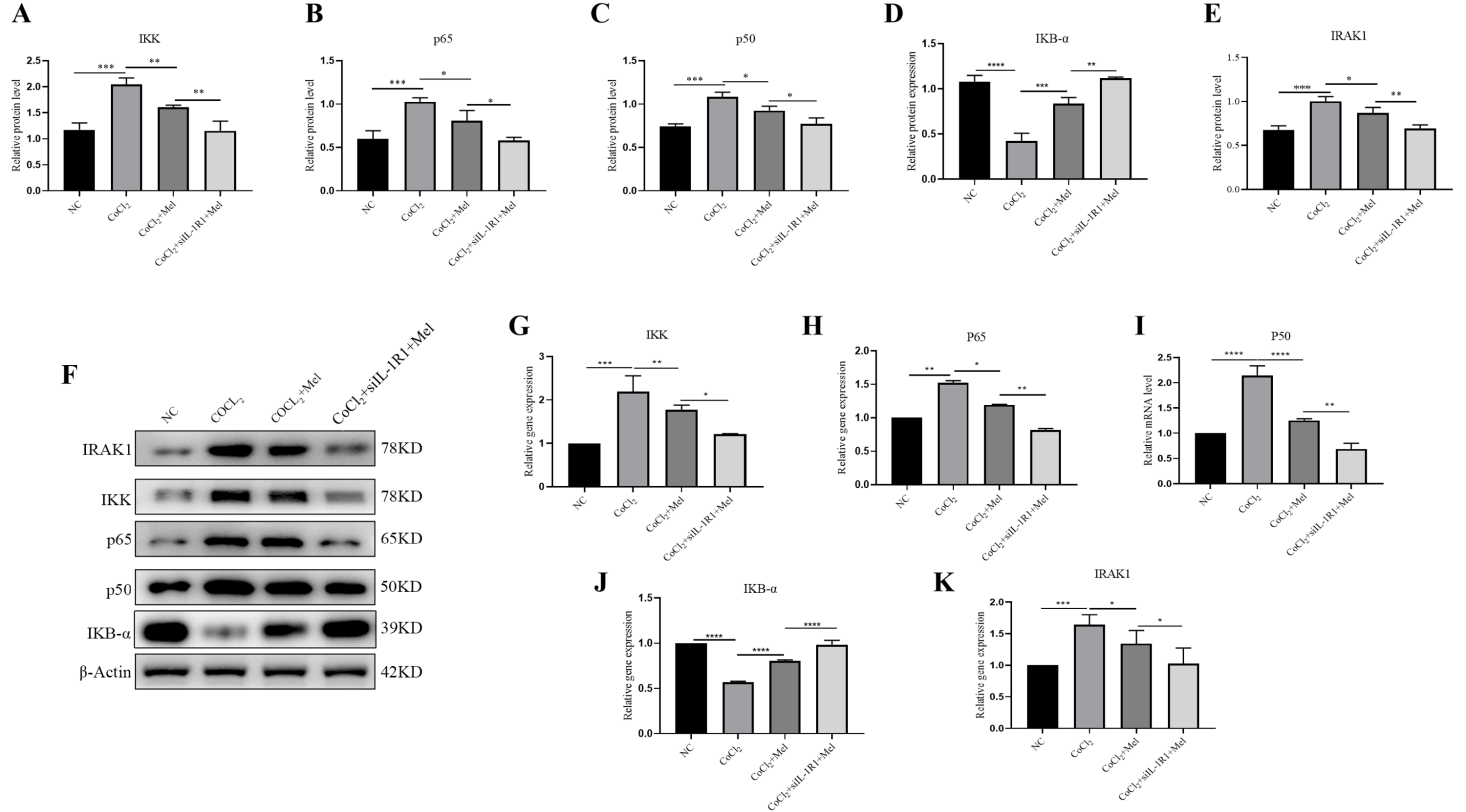
Effect of Mel inhibiting the NF-κB pathway by inhibiting IL-1R1 (A-E) RT-qPCR results showing mRNA expressions of IKK, IKB-α, p65, p60, and IRAK1. (F) Western blot results of protein expression of IKK, IKB-α, p65, p60, and IRAK1. (G-K) Quantitative analyses of IKK, IKB-α, p65, p60, and IRAK1 protein expression. *p < 0.05, **p < 0.01, ***p < 0.001, ****p < 0.0001 versus control and other groups. Data are presented as mean ± standard deviation. RT-qPCR: Quantitative reverse transcription-polymerase chain reaction; IL-1R1: Interleukin-1 receptor type 1; NF-κB: Nuclear factor kappa B.

To understand the regulation mechanism of Mel toward IL-1R1, RT-PCR, and western blot studies were conducted to identify the primary proteins associated with the NF-κB pathway, namely, IKK, IKBα, p65, and p60. The results showed that under hypoxic conditions, the NF-κB pathway was activated. However, treatment with Mel upregulated the expression of IKBα and downregulated the expression of IKK, p65, and p60, and the knockdown of IL-1R1 further enhanced this effect (Figure 4, Panels A-D, F-J). Specifically, the results showed that the treatment with Mel and the knockdown of IL-1R1 downregulated the overexpression of IRAK1 induced by the CoCl_2_-induced hypoxia model (Figure 4, Panels E, K), further confirming that Mel inhibits the NF-κB pathway by reducing IL-1R1 expression. The findings of this study provide further evidence that Mel has a protective effect on injured SCs via the inhibition of IL-1R1 expression.

## Discussion

Er-Rouassi et al. [11] studied the effects of bee venom and its main components on facial nerve injury in mice. In the experiment, the bee venom treatment group showed better behavioral recovery than the phospholipase A2 and control groups. The facial hair movement of the bee venom-treated mice recovered more quickly than the other groups, and the nasal deviation disappeared completely after two weeks of treatment. Pathological study showed that the bee venom treatment group restored normal fluorescent gold labeling of facial motor neurons four weeks after treatment, but this recovery was not observed in the other groups. This result suggests that bee venom and its main components can promote the structural and functional recovery of facial nerve injury in mice. However, this study did not investigate the mechanism of bee venom on the recovery of facial nerve injury.

The onset of IFP and progression of facial nerve injury are closely related to oxidative stress. Epidemiological studies have shown that Bell’s palsy is related to high concentrations of NO_2_ [12]. It was also reported that native thiol and total thiol were significantly lower, whereas disulfide levels were higher in Bell’s palsy patients than in healthy controls [13]. Furthermore, serum total oxidant status activities, oxidative stress index values [14], and malondialdehyde and vitamin E in serum and saliva are significantly increased in patients with Bell’s palsy [15]. The revelation of the high oxidative stress state in patients with IFP may be helpful to clarify its pathogenesis and contribute to improvement in the management or prevention of the disease.

IL-1R1, as a kind of inflammatory factor and transmitter, plays a role in many neurological diseases such as mild closed-head injury, genotype-dependent glioblastoma, postoperative cognitive dysfunction, seizures, and experimental autoimmune encephalomyelitis. IL-1R1 signaling plays a key role in Th17 cell differentiation and the development of autoimmune diseases [16]. In terms of host-pathogen infection (e.g., mycobacterium tuberculosis), the H1N1 influenza virus IL-1R1 also plays an important role. IL-1R1 is required for dendritic cell-mediated T-cell reactivation within the central nervous system in cases of West Nile virus encephalitis. Some mediators (e.g., miRNAs) can significantly inhibit the NF-κB signaling pathway by targeting IL-1R1. Downregulating the activation of the IL-1β/IL-1R1/MyD88/NF-κB pathway can reduce bleomycin-induced lung inflammation and fibrosis in mice, suggesting that the IL-1R1/NF-κB signaling pathway plays an important role in the occurrence and development of inflammation.

Some certain obstacles must be overcome before bee venom therapy is broadly applied clinically. Local injection of bee venom can lead to hyperalgesia, allodynia, and inflammatory responses at the injection site and in the body, which has been confirmed in our long-term practice and animal experiments [17,18]. Due to the sensitization, hemolysis, and cytotoxicity of Mel, its clinical application is limited [19,20]. In the past, people used milk, soap, and other daily smears to treat the pain, redness, and swelling resulting from bee stings, but their effect was uncertain. In recent years, researchers have developed several drugs to treat bee stings. For example, Tender et al. developed the mini-αA-crystallin gel [21], which is used to treat patients with severe bee stings. For modern drugs that must be directly injected into muscles or veins, prevention and mitigation of toxic side effects are particularly important. Fusing Mel with glutathione S-transferase [22], using novel nanoparticle carriers [23-25] or liposomes of hyaluronic acid [26], loading on soluble microneedles [27], and the coupling of N-terminal fatty acid conjugation [28] to develop new Mel-derived lipopeptides represent new ways to reduce toxicity and increase drug effectiveness in recent years. With the progress of science and technology, it is believed that the clinical application and marketization of bee venom and its derivatives will usher in greater development.

However, there are also obvious limitations in this study. For example, in vitro cytology research is based on a relatively single in vitro cell survival environment. The physiological, pathological, and pharmacological procedures are simplified and cannot be equated with the biological process in vivo, so the role of Mel in the internal environment, including in animals and humans, needs further research to confirm which will be the future direction of work. In addition, studies based on the NF-κB pathway are also one of the aspects that affect the occurrence and development of oxidative stress damage caused by hypoxia, so the role of other signaling pathways in oxidative stress damage caused by hypoxia deserves further study. Similarly, the effect of melittin on them is also worth further investigation.

## Conclusions

In summary, our findings in the present study identified that IL-1R1 may play an important protective role in SCs injury in vivo. Mel alleviates oxidative stress injury in SCs by targeting IL-1R1 to downregulate NF-κB-mediated inflammatory response. These results indicate that Mel represents a potential therapeutic measure to effectively limit the progression of IFP.

## References

[1] Kline LB, Kates MM, Tavakoli M (2021). Bell palsy. JAMA.

[2] Psillas G, Antoniades E, Ieridou F, Constantinidis J (2019). Facial nerve palsy in children: a retrospective study of 124 cases. J Paediatr Child Health.

[3] Balchander D, Cabrera CI, Qureshi H (2024). Bell's palsy and COVID-19: insights from a population-based analysis. Facial Plast Surg Aesthet Med.

[4] Yilmaz M, Tarakcioglu M, Bayazit N, Bayazit YA, Namiduru M, Kanlikama M (2002). Serum cytokine levels in Bell's palsy. J Neurol Sci.

[5] Kim MH, Park SY (2021). Population-based study and a scoping review for the epidemiology and seasonality in and effect of weather on Bell's palsy. Sci Rep.

[6] Liu P, Zhou P, Zhang X, Zhao D, Chen H, Hu K (2023). Pterostilbene mediates glial and immune responses to alleviate chronic intermittent hypoxia-induced oxidative stress in nerve cells. PLoS One.

[7] Chidlow G, Wood JP, Casson RJ (2017). Investigations into hypoxia and oxidative stress at the optic nerve head in a rat model of glaucoma. Front Neurosci.

[8] Liu Z, Li X, Zhao Y, Zhao C, Chen C, Li Z, Huo W (2022). Bell’s palsy may associate with increased IL1R1: a pilot novel gene and lncRNA study. Neurol Neurobiol.

[9] Osuru HP, Lavallee M, Thiele RH (2022). Molecular and cellular response of the myocardium (H9C2 cells) towards hypoxia and HIF-1α inhibition. Front Cardiovasc Med.

[10] Zhao L, Qu W, Wu Y, Ma H, Jiang H (2014). Dorsal root ganglion-derived Schwann cells combined with poly(lactic-co-glycolic acid)/chitosan conduits for the repair of sciatic nerve defects in rats. Neural Regen Res.

[11] Er-Rouassi H, Bakour M, Touzani S, Vilas-Boas M, Falcão S, Vidal C, Lyoussi B (2023). Beneficial effect of bee venom and its major components on facial nerve injury induced in mice. Biomolecules.

[12] Kim SY, Min C, Choi J, Park B, Choi HG (2020). Air pollution by NO(2) is associated with the risk of Bell's palsy: a nested case-controlled study. Sci Rep.

[13] Babademez MA, Gul F, Kale H (2017). Thiol/disulphide homeostasis in Bell's palsy as a novel pathogenetic marker. Clin Otolaryngol.

[14] Terzi S, Dursun E, Yılmaz A, Özergin Coşkun Z, Özgür A, Çeliker M, Demirci M (2017). Oxidative stress and antioxidant status in patients with Bell's palsy. J Med Biochem.

[15] Alyassiri AMH, Zaidan TF (2019). Oxidative and antioxidant status in both serum and saliva of patients with idiopathic facial weakness (Bell's palsy). J Med Biochem.

[16] Sha Y, Markovic-Plese S (2011). A role of IL-1R1 signaling in the differentiation of Th17 cells and the development of autoimmune diseases. Self Nonself.

[17] Li KC, Chen J (2004). Altered pain-related behaviors and spinal neuronal responses produced by s.c. injection of melittin in rats. Neuroscience.

[18] Sumikura H, Andersen OK, Drewes AM, Arendt-Nielsen L (2006). Secondary heat hyperalgesia induced by melittin in humans. Eur J Pain.

[19] Gajski G, Domijan AM, Žegura B (2016). Melittin induced cytogenetic damage, oxidative stress and changes in gene expression in human peripheral blood lymphocytes. Toxicon.

[20] Lyu C, Fang F, Li B (2019). Anti-tumor effects of melittin and its potential applications in clinic. Curr Protein Pept Sci.

[21] Tender T, Rahangdale RR, Nampoothiri M, Raychaudhuri R, Mutalik S, Sharma K, Chandrashekar HR (2024). Revamped mini-αA-crystallin showed improved skin permeation and therapeutic activity against melittin-induced toxicity. Toxicon.

[22] Rayahin JE, Buhrman JS, Gemeinhart RA (2014). Melittin-glutathione S-transferase fusion protein exhibits anti-inflammatory properties and minimal toxicity. Eur J Pharm Sci.

[23] Vu HD, Huynh PT, Ryu J (2021). Melittin-loaded iron oxide nanoparticles prevent intracranial arterial dolichoectasia development through inhibition of macrophage-mediated inflammation. Int J Biol Sci.

[24] Ye R, Zheng Y, Chen Y (2021). Stable loading and delivery of melittin with lipid-coated polymeric nanoparticles for effective tumor therapy with negligible systemic toxicity. ACS Appl Mater Interfaces.

[25] Tang S, Zhou L, He H (2022). MnO(2)-melittin nanoparticles serve as an effective anti-tumor immunotherapy by enhancing systemic immune response. Biomaterials.

[26] Li Y, Ruan S, Wang Z, Feng N, Zhang Y (2021). Hyaluronic acid coating reduces the leakage of melittin encapsulated in liposomes and increases targeted delivery to melanoma cells. Pharmaceutics.

[27] Du G, He P, Zhao J (2021). Polymeric microneedle-mediated transdermal delivery of melittin for rheumatoid arthritis treatment. J Control Release.

[28] Huang S, Su G, Jiang S, Chen L, Huang J, Yang F (2024). New N-terminal fatty-acid-modified melittin analogs with potent biological activity. Int J Mol Sci.

